# Partial protoporphyrinogen oxidase (*PPOX*) gene deletions, due to different *Alu*-mediated mechanisms, identified by MLPA analysis in patients with variegate porphyria

**DOI:** 10.1186/1750-1172-8-13

**Published:** 2013-01-16

**Authors:** Michela Barbaro, Maire Kotajärvi, Pauline Harper, Ylva Floderus

**Affiliations:** 1 Department of Molecular Medicine and Surgery, Karolinska Institutet, Stockholm, Sweden; 2 Centre for Inherited Metabolic Diseases, Karolinska University Hospital, Stockholm, Sweden; 3 Porphyria Centre Sweden, Karolinska University Hospital, Stockholm, Sweden; 4 Department of Laboratory Medicine, Division of Metabolic Diseases, Karolinska Institutet, Stockholm, Sweden; 5 CMMS L7:05, Karolinska University Hospital, SE-171 76, Stockholm, Sweden

**Keywords:** *Alu*-mediated deletions, Protoporphyrinogen oxidase, *PPOX*, VP, Variegate porphyria, MLPA

## Abstract

Variegate porphyria (VP) is an autosomal dominantly inherited hepatic porphyria. The genetic defect in the *PPOX* gene leads to a partial defect of protoporphyrinogen oxidase, the penultimate enzyme of heme biosynthesis. Affected individuals can develop cutaneous symptoms in sun-exposed areas of the skin and/or neuropsychiatric acute attacks. The identification of the genetic defect in VP families is of crucial importance to detect the carrier status which allows counseling to prevent potentially life threatening neurovisceral attacks, usually triggered by factors such as certain drugs, alcohol or fasting.

In a total of 31 Swedish VP families sequence analysis had identified a genetic defect in 26. In the remaining five families an extended genetic investigation was necessary. After the development of a synthetic probe set, MLPA analysis to screen for single exon deletions/duplications was performed.

We describe here, for the first time, two partial deletions within the *PPOX* gene detected by MLPA analysis. One deletion affects exon 5 and 6 (c.339-197_616+320del1099) and has been identified in four families, most probably after a founder effect. The other extends from exon 5 to exon 9 (c.339-350_987+229del2609) and was found in one family. We show that both deletions are mediated by *Alu* repeats.

Our findings emphasize the usefulness of MLPA analysis as a complement to *PPOX* gene sequencing analysis for comprehensive genetic diagnostics in patients with VP.

## Introduction

Variegate porphyria (VP, OMIM 176200) is an autosomal dominant disease caused by mutations in the protoporphyrinogen oxidase (*PPOX*) gene, causing a partial defect of the seventh catalytic step of the haem biosynthetic pathway. The enzyme protoporphyrinogen oxidase (PPOX) [EC 1.3.3.4] may become overloaded under conditions with high demand of hepatic haem biosynthesis leading to the accumulation of phototoxic and/or neurotoxic metabolites. Thus VP is a mixed form of cutaneous and acute porphyria. Overt disease is associated with skin photosensitivity and/or neurogenic symptoms as acute attacks of severe abdominal pain, autonomic disturbance, motor neuropathy and more rarely even psychiatric symptoms [1].

The clinical penetrance of VP is about 40% [2,3] and symptoms almost never manifest before puberty. Skin symptoms are manifest as skin fragility and blistering in sun exposed areas of the body, and are caused by the accumulation of protoporphyrinogen (the substrate of PPOX) in the skin. The skin symptoms are the same as those presented in porphyria cutanea tarda (PCT) and hereditary coproporphyria (HCP). The neurovisceral symptoms are undistinguishable from those for acute intermittent porphyria (AIP) or HCP. Surplus protoporphyrinogen may cause an allosteric inhibition of porphobilinogen deaminase (PBGD, the third enzyme in the haem biosynthetic pathway) leading to accumulation of the neurotoxic metabolites 5-aminolevulinic acid (ALA) and porphobilinogen (PBG) [4].

During overt disease the diagnosis of VP is based on clinical symptoms and biochemical laboratory investigations. The porphyrin biochemical pattern characteristic for VP is the presence of a plasma fluorescence marker with a maximum peak at 624 – 627 nm, increased faecal protoporphyrin and inversed coproporphyrin isomer ratio (i.e. copro III isomer > copro I isomer; normal I > III). The urinary porphyrin metabolite pattern may be normal or abnormal with increased excretion of ALA, PBG and coproporphyrin depending on the degree of the metabolic overload of the haem biosynthetic pathway. Erythrocyte porphyrin concentration is usually normal. The activity of the PPOX enzyme, located in the mitochondrial membrane, is measured in lymphocytes or fibroblasts [4]. Further DNA investigations are usually required in order to easily detect asymptomatic/latent relatives [5]. The detection of carriers is of extreme importance and VP patients or latent carriers, as in all acute porphyrias, shall be encouraged to avoid porphyrogenic agents as e.g. certain drugs, alcohol and low carbohydrate diets etc. [6,7].

The *PPOX* gene is located on chromosome 1q22-23, has a size of 5.5 kb and contains 13 exons, one non-coding and 12 coding that encode the 477 amino acid PPOX enzyme.

The prevalence of VP in Sweden has been estimated to be 1:100 000 and the mutation pattern in fourteen families has been reported [5]. Before the present study a total of 20 different mutations have been found among the Swedish VP patients (unpublished data). Homozygous or compound heterozygous mutations, with seriously comprised phenotype since early childhood, have been described [8-10] but no such cases have been identified in Sweden.

In five families with well established clinical and biochemical VP diagnosis, the underlying gene defect was not detected by routine DNA sequencing analysis. The aim of this study was to develop a MLPA probe set to screen for possible deletions/duplications affecting the *PPOX* gene in these families.

## Patients and methods

### Patients

**Figure 1:**
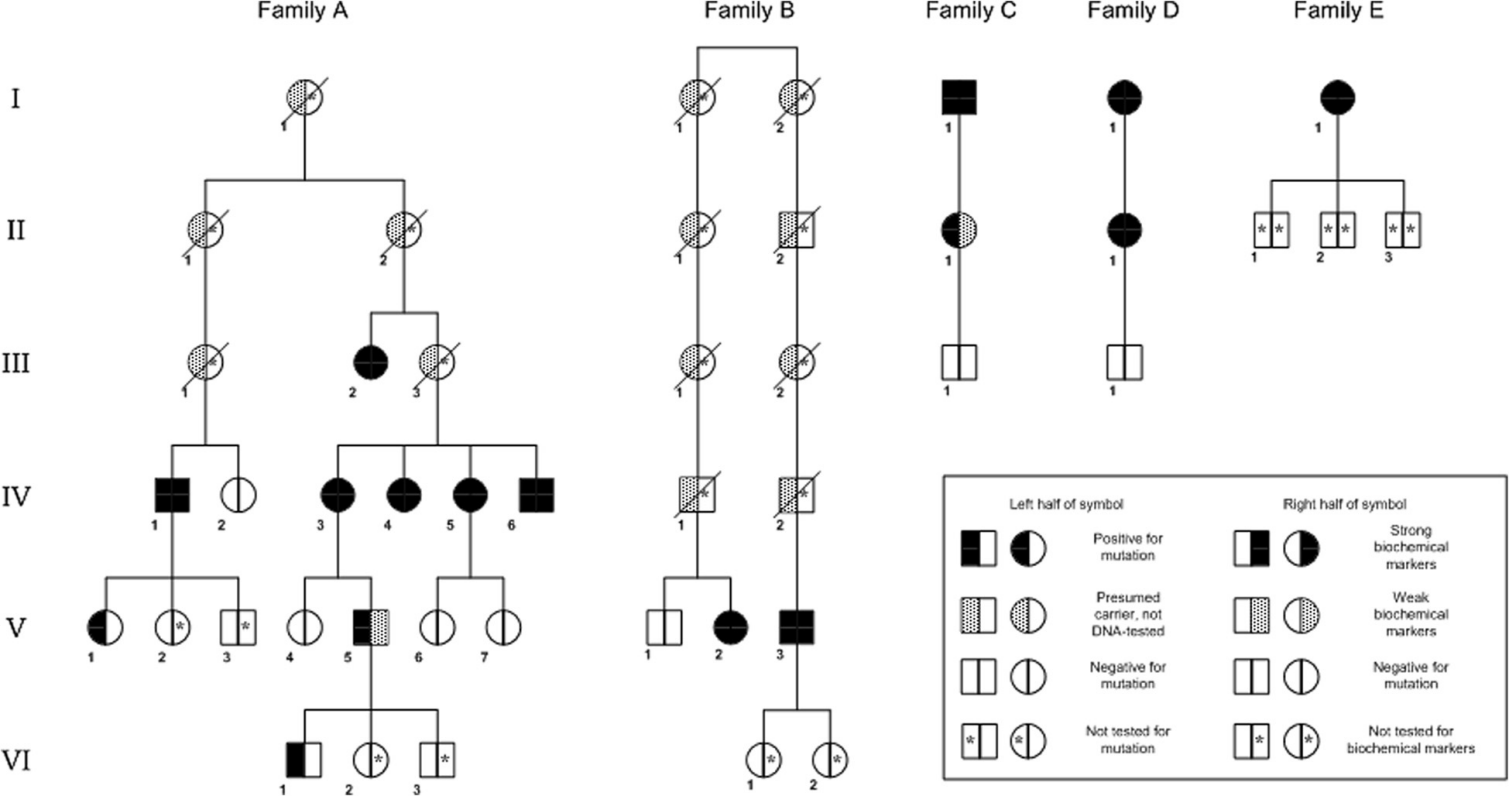
**Pedigrees of VP families carrying partial *PPOX *deletion.** Pedigrees of the five families carrying partial *PPOX* deletion with DNA results and markers for biochemical symptoms. In families **A-D** affected members carry an exon 5–6 deletion. In family E the patient carries an exon 5–9 deletion. Members in family **A-D** for whom neither biochemical nor genetic investigation were performed are omitted for simplification.

Patients, biochemically confirmed to be affected by VP, in which *PPOX* routine analysis by PCR and sequencing had failed to identify a disease causing mutation have been selected for this study. The patients included in this study belong to five apparently unrelated Swedish families (Figure 1). The genetic analysis, that includes 845 nucleotides of the promoter region and the non-coding exon 1 of the *PPOX* gene, as well as the biochemical investigations used to identify patients and latent carriers have been previously described [5].

The study was approved by the Ethics Committee of Karolinska Institutet, no. 395/00.

### DNA

Genomic DNA was extracted from peripheral blood samples by the QIAamp DNA Mini kit (QIAGEN, Hilden, Germany), according to the manufacturer’s protocol.

### MLPA probe set development and analysis

The target sequence of each synthetic half probe was designed according to the recommendations by Stern et al. [11]. One probe pair for each exon of the *PPOX* gene was designed, including the first non-coding exon. In the probe set three internal control probe pairs and a pilot probe pair were included [12] as well as a probe targeting the *SRY* gene on the Y chromosome to control the correct order of sample loading (see Table 1). MLPA reactions were performed as previously described [12], starting from 100 ng of genomic DNA and using the reagents and the recommendations of the EK1 MLPA reagent kit (MRC-Holland, Amsterdam, The Netherlands). The PCR products were separated by capillary electrophoresis on an ABI 3130XL Genetic Analyzer (Applied Biosystems, Warrington, UK). Trace data were analyzed using the Gene Mapper v4.0 software (Applied Biosystems), and the integrated peak areas and heights were exported to an Excel spreadsheet (Microsoft, Silicon Valley, CA). For each sample, the peak heights were first normalized to the average peak height of the control probes followed by normalization to the average peak height of the control samples included in the run. The sample run was considered acceptable if the ratio for the internal control probe pairs was between 0.8 and 1.2. Threshold values for deletion and duplication were set at 0.75 and 1.25, respectively.

**Table 1:** PPOX probe mix

**Probe name**	**Size**^**a**^	**5**^**′**^**half hyb sequence**^**b**^	**3**^**′**^**half hyb sequence**^**c**^	**SD**	**N1**	**N2**	**Fam A**	**Fam E**
GABRA4^d^	84	CAGCCTGTTGTCATAACCATCG	AGCAAACTGTCCAGGATGCG	0.20	nd	nd	nd	nd
RELN^e^	87	CAGCATTACGGAATGAAGGTCA	CCACAAGAAGTGGCTTCACAACC	0.04	1.02	0.99	0.98	0.96
PPOXex1	90	GGGAGTAGCGGATTTGAAGCACTT	GTTGGCCTACAGAGGTGTGGCAAG	0.03	0.94	1.00	1.07	1.01
PPOXex7v3	93	CTGAGCGCTGGAGCCAGTGGTCACTT	CGTGGAGGTCTAGAGATGTTGCCTC	0.04	1.00	0.99	1.03	**0.55**
PPOXex5v5	96	GCAAAGAGCCTGATGAGACTGTGC	ACAGTTTTGCCCAGCGCCGCCTTGGACCTG	0.03	1.04	1.00	**0.57**	**0.60**
PPOXex6	99	GTGTTTGCAGGCAACAGCCGTGAGCTCA	GCATCAGGTCCTGCTTTCCCAGTCTCTTC	0.03	1.02	1.02	**0.59**	**0.58**
PPOXex3	102	GCTCCGTTCGAGGCCCTAATGGTGCTATCT	TTGAGCTTGGACCTCGGGGAATTAGGCCAG	0.02	1.00	0.93	1.04	1.11
CLDN16^**e**^	105	GACACAAGGGTGTAAAATGCACG	TTTCAGGGTGTGTTTGCATATGATTTAATCAATCAGTATG	0.04	1.00	1.02	1.01	1.00
PPOXex9	108	GTGCCATCACTGCAGTGTCTGTAGCTGTGGTGA	ATCTGCAGTACCAAGGAGCCCATCTGCCTGTCC	0.03	1.05	0.99	1.02	**0.58**
PPOXex11	111	CTGTTTCAACAGCGGGCCCAGGAAGCAGCTGCTA	CACAATTAGGACTGAAGGAGATGCCGAGCCACTGC	0.03	1.04	1.04	0.99	1.22
PPOXex4	114	CATGCCCTACCCACTGGCCTCAGGTAACACCAGCA	CCTCCGCTCCTTTTACTGTGCCCTCATCCTCATATGC	0.02	1.05	1.03	1.06	1.16
PPOXex8	117	CTCATTTTCTGGGTCTCTCAAATGTTTTCATGCTCTC	AGGTATCTCTAAGGGACAGCAGTCTGGAGGCTGACCAC	0.04	1.06	1.03	0.98	**0.56**
PPOXex12	120	CTGGCAAAAACTAGGTAAGTTGGGAAAACAGCTGGGCT	GAGGAGGGCCAAGGACATCAGACCCCCAGCTAAAACATTC	0.03	1.06	1.09	0.99	1.16
PPOXex10	123	CTGACGCATGAATGTCCTTCTCTCCAGGGATTTGGACATT	TGGTGCCATCTTCAGAAGATCCAGGAGTCCTGGGAATCGTG	0.03	1.06	1.09	0.93	1.16
PPOXex13	126	GCTGTTAATGACTGTATAGAGAGTGGGCGCCAGGCAGCAGTCA	GTGTCCTGGGCACAGAACCTAACAGCTGATCCCCAACTCTC	0.03	1.08	1.03	1.04	1.13
RB1ex23^**e**^	129	GTCACCAATACCTCACATTCCTCGAAGCCCTTACAAGTTTCCT	AGTTCACCCTTACGGATTCCTGGAGGGAACATCTATATTTCACC	0.02	1.00	1.02	0.98	1.11
PPOXex2v4	132	CCTAAGGTGAGTGCTCCACTTGTGCCAGAGGGAGCTTCATTTAATGC	TCTTCCCATTTCCATCAAAAGCTAGATGGATCCTGGCCCTCTG	0.02	1.01	1.00	1.09	1.08
SRY	135	CAGTGCAAAGGAAGGAAGAGCTTCTCCGGAGAGCGGGAATATTCT	CTTGCACAGCTGGACTGTAATCATCGCTGTTGAATACGCTTAACATAG	nd	nd	nd	nd	nd

^a^Size of the ligaton product in bp.

^b^The 5^′^ half-probes are preceded by the universal tag sequence GGGTTCCCTAAGGGTTGGA.

^c^The 3^′^ half-probes are followed by the universal tag sequence CTAGATTGGATCTTGCTGGCAC and are phosphorylated at the 5^′^ end.

^d^Pilot probe.

^e^Reference probes.

*SD* standard deviation.

*Nd* not determined.

### Deletion breakpoint characterization

Amplifications of the deletion junctions were performed using a forward primer within intron 3 (5^′^-TTGGTGGGTCAGATCTTC-3^′^) in combination with a reverse primer within intron 7 (5^′^-CACTGACAGGTTCATTACAC-3^′^) for the exon 5–6 deletion or a reverse primer within intron 10 (5^′^-CCTAAGGAGGGAATATAGCACTG-3^′^) for the exon 5–9 deletion. PCR was carried out in a 50 μl reaction mixture containing 100 ng genomic DNA, 0.4 μM of each primer, 200 μM of each dNTPs, 1X PCR buffer F514L (Finnzyme, Espoo, Finland) and 0.5 U DyNAzyme EXT DNA polymerase (Finnzyme). After an initial denaturing step at 94°C for 2 min, 39 cycles, consisting of denaturation at 94°C for 30 sec, annealing at 57°C for 30 sec and elongation at 70°C for 1 min, were carried out, followed by a final elongation step at 70°C for 10 min. A total of 45 μl of PCR products were purified with the Agentcourt^® ^Ampure kit (Beckman Coulter, Brea, CA).

Sequencing reactions of PCR products were performed using the ABI Big Dye Terminator v3.1 kit (Applied Biosystems) followed by ethanol precipitation in a 96-well plate according to manufacturer’s protocol, using the same primer as used for the PCR reaction. Sequence reactions were separated by capillary electrophoresis in an ABI 3130XL Genetic Analyzer (Applied Biosystems) and electropherograms were visualized by Sequence Scanner v1.0 (Applied Biosystems).

### Haplotyping

By routine sequencing analysis of the *PPOX* gene in some members of the four families we determined an intragenic SNP haplotype of the allele carrying the exon 5–6 deletion. The SNPs considered are the validated SNPs from dbSNP [13] within the coding sequence; rs2301286 and rs72714915 within the 5^′ ^UTR and rs2301287 within intron 2 have also been included as data were available from routine analysis and they are quite variable within the Swedish population (Table 2).

**Table 2:** Haplotypes in the allele with the exon 5–6 deletion

**SNP**	**SNP alleles**	**Location**	**Average Heterozygosity**	**MAF**	**Fam A**	**Fam B**	**Fam C**	**Fam D**	**Shared haplotype**	**Alleles in Swedish population**
rs2301286	A/C	Exon 1 UTR	0.397	A = 0.3832	C	C	C	C	**C**	A/C
rs115158839	G/T	Exon 1 UTR	0.033	T = 0.006	G	G	G	G	**G**	G
rs72714915	C/G	Exon 1 UTR	0.095	G = 0.0367	C	C	C	C	**C**	C/G
rs2301287	C/G	Intron 2	0.444	G = 0.3759	C	C	C	C	**C**	C/G
rs12030747	G/A	Exon 7 (Glu234Lys)	0.013	NA	G	G	G	G	**G**	G
rs36013429	G/A	Exon 9 (His304Arg)	0.008	A = 0.0476	G	G	G	G	**G**	G/A

*MAF* minor allele frequency.

*NA* not available.

## Results

### MLPA analysis

The performance of the PPOX probe set (Table 1) was tested using DNA samples from 11 normal controls. All probes presented a SD <10%, thus proving to have a reliable and sufficiently consistent performance.

**Figure 2:**
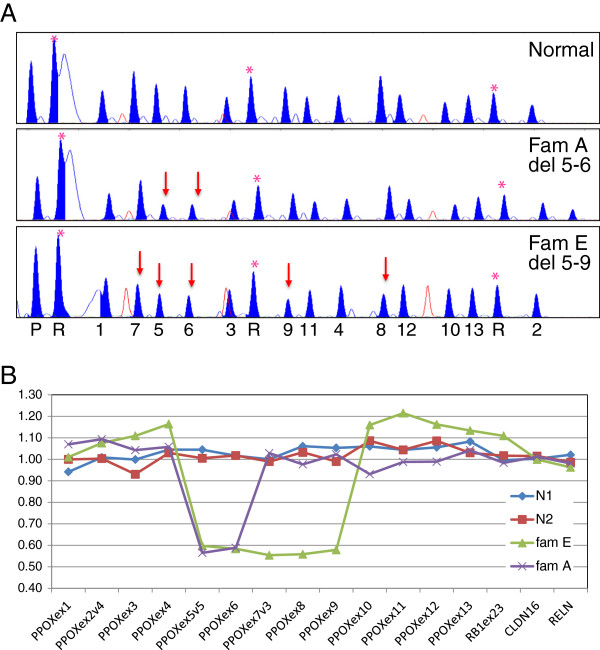
**PPOX probe set MLPA results. **(**A**). MLPA trace results for the PPOX probe set in patients belonging to VP families **A** and **E**, and two normal controls (N1 and N2) used as reference. Peaks corresponding to control probes are labeled (*). On the X-axis at the bottom of the figure the numbers indicate the *PPOX* exon to which the peak corresponds; P the pilot probe; R the reference probes. Red arrows indicate the deleted probes. (**B**). Graph showing the analysis results from Excel. Probes on the *PPOX* gene are ordered according to their location along the gene. Internal reference probes are included and show a ratio value near 1. The ratio values near 0.5 indicate a heterozygous deletion for the corresponding probes.

At least one affected member for each family was analyzed by MLPA. Patient samples belonging to families A, B, C and D showed ratio values consistent with a heterozygous deletion for the probes within exons 5 and 6 (Figure 2). The sample belonging to the patient in family E showed a deletion extending from exon 5 to exon 9 (Figure 2).

To identify all the carriers of the deletions, all available family members were analyzed by MLPA or PCR; the family trees in Figure 1 summarize the results obtained.

### Deletion characterization

To confirm and fine map the deletions, the deletion junctions were amplified and sequenced. Using a forward primer within intron 3 and a reverse primer within intron 7, the PCR product in a normal control gave a band of 1904 bp, while in the samples with the exons 5–6 deletion a band of approximately 1 kb was obtained. By sequencing we could also establish that in all four families the affected members share the same deletion of 1099 bp. According to the Human Gene Variation Society [14] the deletion can be described as c.339-197_616+320del1099bp, using the reference sequence NM_000309.3.

In family E the PCR product in the patient had a size of approximately 0.7 kb instead of the wild type size of 3305 bp. By sequencing we determined that the deletion has a size of 2609 bp, thus it can be described as c.339-350_987+229del2609bp.

### Haplotype analysis

Patients belonging to the four different families with the exon 5–6 deletion share, on the allele with the deletion, the same haplotype for the 6 SNPs considered (Table 2).

## Discussion

VP is caused by mutations in the *PPOX* gene. All the 150 mutations reported so far are point mutations or small insertions or deletions [15] with the largest deletion being 23 bp long [16], all of them detectable by a PCR and sequencing approach. In our patient population we had failed to identify a disease causative mutation in five families by routine sequence analysis. We have therefore developed a MLPA probe set to screen for deletion/duplication of each exon of the *PPOX* gene that would escape detection by PCR and sequencing.

We identified two deletions, one affecting exon 5 and 6 and the other extending from exon 5 to exon 9. These are the first partial deletions of the *PPOX* gene described so far.

**Figure 3:**
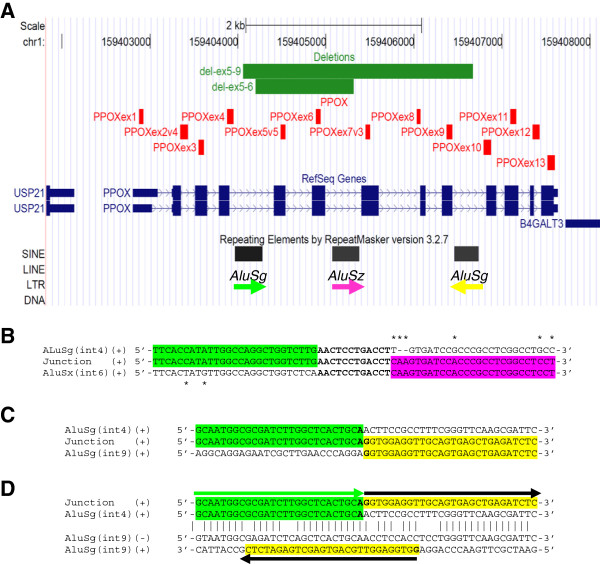
**PPOX MLPA probe set and deletion characterizations. **(**A**). Representation from the UCSC genome browser (Mar2006. NCBI36, hg18) of the protoporphyrinogen (*PPOX)* gene locus on 1q22-23. Probes included in the 'PPOX MLPA probe set' are represented as vertical red lines. Deletions are represented by horizontal green lines. Repeats identified by Repeat Masker are represented by black boxes, arrows underneath indicate the respective genomic orientation. The colour of the arrow is used to highlight the sequence of the corresponding *Alu* in the sequence analysis showed below. (**B**). Sequence analysis of the exon 5–6 deletion junction. Partial alignment of the *AluSg* within intron 4, the deletion junction sequence and the *AluSx* within intron 6. Single nucleotide differences between the two *Alu* repeats are denoted by an asterisk (*). Nucleotides in the region of complete homology are bolded. (**C**). Sequence analysis of the exon 5–9 deletion junction. Partial alignment of the deletion junction with the *AluSg* within intron 4 and within intron 9, respectively. The two nucleotides at the junction site are bolded. (**D**). Partial alignment between the *AluSg* within intron 4 and the *AluSg* within intron 9, showing the homology between the two *AluSg* and their inverted orientation. Vertical lines denote identical nucleotides; (+) and (−) indicate the positive and negative filament, respectively. The junction sequence and the positive filament of intron 9 *AluSg* are shown together with arrows to facilitate the understanding of the rearrangement.

Both deletions are mediated by *Alu* sequences. For the exon 5–6 deletion, *in silico* analysis identified a 308 bp *AluSg* element within intron 4 and a 353 bp *AluSz* element within the intron 6 that share 80% identity, according to BLASTN alignment [17]. Sequencing of the deletion junction identified the breakpoint within a 12 bp interval of complete homology (Figure 3). As the two *Alu* elements belong to two different subfamilies and do not share a long stretch of extremely high identity required for homologous recombination, an *Alu*-mediated non-allelic homologous recombination mechanism (NAHR) is unlikely to be responsible for the deletion. An *Alu*-specific microhomology-mediated deletion is the most likely mechanism [18]. Since we found the same deletion in four independent families we further collected pedigree information and analyzed the gene haplotype to evaluate if this deletion occurred independently due to a recurrent deletion mechanism or if there was a possible founder effect. Patients belonging to the four different families share the same intragenic SNP haplotype on the allele with the exon 5–6 deletion. Furthermore, the oldest family members who are potential carriers of the mutation were born in three neighbouring parishes in the South East of Sweden. Even if we could not find a common ancestor between them, it is very likely that they are related, thus suggesting a founder effect for this mutation rather than a recurrent deletion mechanism. This could represent an example of clan genomics: a recently developed mutation of clinical relevance spreading in a specific population [19].

For the exon 5–9 deletion, *in silico* analysis determined that the 308 bp *AluSg* element within intron 4 share also 87% identity with a 278 bp *AluSg* located within intron 9 with an inverted orientation. Sequencing of the breakpoint region doesn’t show a minimal region of homology. It is thus impossible to discriminate if this deletion is mediated by a recombination based mechanism such as NHEJ (non-homologous end joining ) or by a single event of a replication-based mechanism like FoSTeS (Fork Stalling and template switching) or MMBIR (microhomology-mediated break-induced replication) [18,20].

*Alu*-mediated genomic rearrangements have been shown to be involved in 0.3% of human genetic disease [21] where they can be responsible for both recurrent and non-recurrent rearrangements [22]. Interestingly the *AluSg* within intron 9 shares 80% identity and has inverted orientation not only with respect to the *AluSg* within intron 4, where it is involved in the exon 5–9 deletion, but also with respect to the *AluSz* within intron 6. As inverted *Alu* repeats are a threat to genome stability [23], *PPOX* can be considered at risk for more genetic rearrangements.

Most of the adults positive for the exon 5–6 deletion have presented with biochemical markers indicating VP, mainly typical plasma fluorescence marker and increased faecal porphyrins, with dominance of coproporphyrin III isomer. Clinical symptoms such as skin blisters and/or neuropsychiatric symptoms have been present in several patients and some have previously been misdiagnosed as PCT or AIP, Patient A IV:4 had at her fourth decade of life suffered from severe neuropsychiatric symptoms, abdominal pain, motor neuropathies and periods of hallucinosis. She has later in life developed skin blisters. Patient B V:2 presented with abdominal pain and neurological symptoms at the age of 21 and had an AIP diagnosis at 24. During the fourth decade of life she presented skin symptoms and was diagnosed as PCT. At present she is asymptomatic but has a chronic hypertension and decreased kidney function. Patient D II:1 at the age of 30 had skin lesions resembling PCT and during anaesthesia (caesarean) she developed paralysis of facial muscles. Patient C I:1 was diagnosed at the age of 48 with PCT and later developed as well periods of abdominal pain, depression and mild psychosis. VP diagnosis was lately biochemically confirmed at the age of 77. At that time he had normal excretion of urinary haem precursors but several-fold increased faecal porphyrins and a distinct fluorescence marker in plasma (627 nm). He died at the age of 88 years. His daughter was identified by genetic analysis at the age of 55. She has been clinically latent and has weak biochemical markers. Two younger carrier members in Family A (V:1 and VI:1) at the age of 26 and 14 respectively, show neither biochemical signs nor clinical symptoms yet. The patient E I:1 with the exon 5–9 deletion was diagnosed at age 27 after a period of abdominal pain. She has also had temporary skin symptoms. At present she has hypertension and end stage renal disease.

Despite sharing the same mutation these patients present extensive phenotype variation and early diagnosis is of main importance to prevent outbreak of clinical symptoms. Acute porphyria carriers above the age of 50 should be included in yearly liver screening programme including radiological investigation in order to detect primary liver cancer, a known common complication in acute hepatic porphyria [24-27].

Gene dosage analysis has earlier been shown to increase the diagnostic sensitivity for the two other acute porphyrias (AIP and HCP) [28] and large deletions have been reported for the *HMBS* and *CPOX* genes, respectively [28-30]. We describe here two partial deletions within the *PPOX* gene, indicating that partial gene deletions should be considered in the genetic diagnosis of all acute porphyrias. The MLPA probe set we have developed represents a useful tool for the identification of such mutations, and should be performed to complement *PPOX* sequencing analysis.

## Competing interest

The authors declare that they have no competing interests.

## Authors’ contributions

MB has done the experimental planning, developed the MLPA probe kit, analysed and interpreted the genetic results and written the manuscript. MK has assisted in developing the MLPA probe kit, has done the experimental laboratory work and analysed the results. PH has collected the patients, written and evaluated the clinical characterizations. YF has done the experimental planning, collected and evaluated all patient data and written the manuscript. All authors have read and approved the final manuscript.
